# Deflection Control of Concrete Wide Beams Supporting Columns Using CFRP Composites and Honeycomb Plates

**DOI:** 10.3390/polym17182560

**Published:** 2025-09-22

**Authors:** Abdulaziz Baatiah, Hussein Elsanadedy, Aref Abadel, Husain Abbas, Tarek Almusallam, Yousef Al-Salloum

**Affiliations:** Chair of Research and Studies in Strengthening and Rehabilitation of Structures, Department of Civil Engineering, College of Engineering, King Saud University, P.O. Box 800, Riyadh 11421, Saudi Arabia; 447105717@student.ksu.edu.sa (A.B.); helsanadedy@ksu.edu.sa (H.E.); aabadel@ksu.edu.sa (A.A.); habbas@ksu.edu.sa (H.A.); musallam@ksu.edu.sa (T.A.)

**Keywords:** strengthening, deflection control, CFRP, honeycomb, RC wide beam, planted column, flexural performance

## Abstract

In the Middle East, RC joist slab systems with wide beams are widely used for residential floors. However, when these beams support planted columns, excessive deflection beyond code limits is often observed, despite adequate flexural and shear design. This paper experimentally assesses, for the first time, the efficacy of using carbon-fiber-reinforced polymer (CFRP) sheets alone versus a novel hybrid system comprising CFRP sheets and CFRP/honeycomb plates in controlling deflection in RC wide beams with planted columns. Four RC wide beam specimens at half-scale, each featuring a planted column, were tested to failure. Two control specimens, the first one was designed to reflect standard construction practices. It was sufficiently designed in flexure and shear, but its deflection exceeded code requirements. The second was designed to satisfy the code deflection requirements. The remaining specimens were strengthened using two different techniques: one with externally bonded CFRP sheets and the other with the hybrid system. The findings demonstrated a marked improvement in the flexural performance of the retrofitted wide beams, with peak load increases of 65–71%, stiffness gains of 63–67%, and reduced deflections meeting serviceability requirements (deflection at peak load was reduced by 45–48%). Furthermore, an analysis procedure was developed to estimate the flexural strength and deflection of these beams.

## 1. Introduction

**Figure 1:**
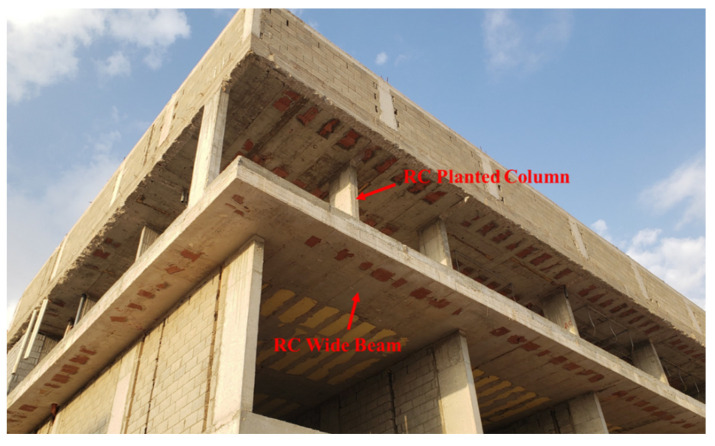
Typical RC wide beam with planted column in residential building in Saudi Arabia (photo taken by Abdulaziz Baatiah).

The joist slab system, comprising concrete wide beams, infill bricks between ribs, and a concrete overlay, is a commonly adopted flooring solution in residential construction across the Middle Eastern region [1]. Figure 1 shows a typical residential building in Saudi Arabia with a reinforced concrete (RC) wide beam having a planted column. The RC wide beams are typically characterized by their shallow depth (<350 mm) and a cross-sectional ratio of width-to-depth exceeding two [2]. This is because of its constructional and architectural advantages. Furthermore, wide beams are used in cases where a limited floor depth is required, while also offering advantages such as a simplified formwork, ease of reinforcement placement, and reduced overall construction cost.

However, in non-seismic regions, architectural demands often lead to the provision of planted columns on these beams. Such columns transfer vertical loads onto the wide beam [3], which subsequently channels these forces to the primary columns below. The application of such columns on shallow wide beams has become a prevalent practice in the Middle East, prompting a critical examination of the behavior of such structures.

The use of planted columns, while practical, could potentially introduce structural issues if not meticulously calculated and implemented. The risk ranges from affecting the building’s serviceability due to excessive deflection to potentially life-threatening scenarios due to partial collapse. In the case of RC wide beams, critical parameters such as deflection and flexural capacity must be carefully evaluated when a planted column is present. Notably, a significant number of RC structures in the Middle East were built using wide beams that no longer meet current structural standards [1]. Accordingly, the adverse effects associated with planted columns can be addressed by strengthening the supporting wide beams.

In recent years, fiber-reinforced polymer (FRP) has been utilized for strengthening deficient RC buildings, especially carbon FRP (CFRP), owing to their superior material characteristics. CFRPs offer significantly higher strength and stiffness-to-weight ratios compared to the commonly used steel plates. Additionally, their noncorrosive nature contributes to enhanced durability, making them particularly effective in structures exposed to aggressive environmental conditions [4,5,6,7,8,9,10]. Among the various CFRP strengthening techniques, the most widely adopted technique involves externally bonding CFRP laminates to the bottom side of flexural members with the help of adhesives [11].

Another strengthening material that was used to upgrade RC beams in flexure is prefabricated composite plates comprised of a honeycomb core covered with FRP face sheets [12]. They are comprised of two FRP composite facings linked to a less stiff, less rigid lightweight core structure between them. The facings and core are structurally bonded by an adhesive, ensuring a seamless connection throughout the depth of the panel. This composite material is employed due to its beneficial properties, including high tensile strength and stiffness, excellent fatigue resistance, corrosion resistance, and relatively high thermal stability [13,14,15,16,17].

A large number of research studies have been conducted to upgrade the bending strength of conventional RC beams, those with depths exceeding their widths, with the help of FRPs [18,19,20,21,22,23,24,25,26,27,28,29,30,31,32]. More recently, the use of honeycomb sandwich panels for strengthening such beams has gained increasing interest among both researchers and practitioners, due to their potential to improve structural capacity and stiffness [12,33,34,35,36]. Consequently, this technique has attracted significant attention within the structural strengthening industry. Among the limited studies addressing the retrofitting of RC wide beams against bending, the study [37] reported that strengthening wide beams with CFRP plates caused a 77% increase in peak load and a 50% decrease in immediate deflection, although long-term deflection reduced by only 13%. In a separate study, Abass and Hassan [38] evaluated three RC wide beams retrofitted using bonded CFRP sheets of varying widths and confirmed that CFRP sheets were able to substantially enhance the strength as well as the stiffness. Furthermore, Al-Negheimish et al. [1] examined the influence of different quantities and configurations of CFRP sheets on the bending behavior of retrofitted RC wide beams. The authors reported that stiffness improvement was directly related to the axial tension stiffness of the CFRP sheets, regardless of their type. It was also observed that distributing an equal amount of FRP in either one or two layers had no significant effect on the beams’ flexural performance. Unlike standard beams, wide beams in joist slab systems typically have lower depth-to-span ratios, and less transverse reinforcement, making them more flexible and susceptible to excessive deflections. Moreover, the addition of a planted column further aggravates serviceability issues and long-term deflection concerns. Therefore, addressing the deflection behavior of RC wide beams with planted columns represents a distinct research need, as existing studies on FRP strengthening mainly focus on conventional beams without accounting for these unique serviceability demands.

Lee and Eom [39] studied measures for meeting shear demand in dapped-end RC beams, considering the effects of shear reinforcement types, such as distributed densified stirrups, hanger reinforcement, and diagonal reinforcement. Distributed stirrups in dapped-end beams provide shear capacity comparable to conventional hanger reinforcement, offering a simplified alternative to reduce reinforcement congestion. Elansary et al. [40] demonstrated that spiral lateral reinforcement improves the shear capacity and ductility of wide beams. Moubarak et al. [41] proposed a novel method using inserted fasteners, which enhanced shear strength by 32–72% and shifted failure modes to flexure. Liu et al. [42] reviewed the bolted side-plating (BSP) technique, highlighting its effectiveness in improving both flexural and shear performance and proposing an analytical design model.

Though the use of wide beams supporting columns is prevalent in the Middle East’s RC framed buildings, such beams were adequately designed to meet flexural and shear requirements. Nevertheless, their design did not include deflection checks, leading to service deflections that exceeded the permissible limits specified by design codes. To maintain the serviceability of such buildings, strengthening is considered essential. Only some research studies have addressed the flexural strengthening of RC wide beams. Even though recent studies [25,43,44] have addressed the stiffness enhancement of RC beams through the addition of FRP composites on the tension side, none have investigated RC wide beams with planted columns. To date, no research has been identified in the accessible literature that specifically investigates the retrofitting of RC wide beams for the purpose of deflection control. The aim of this research is to experimentally examine the retrofitting of such beams featuring planted columns to increase their stiffness for controlling the deflection to values below the codified thresholds. For achieving this purpose, CFRP sheets alone, in comparison with a hybrid composite system comprised of CFRP sheets along with CFRP/honeycomb plates, were utilized for strengthening of these beams. Four specimens were constructed with a planted column at mid-span and then tested to failure. Two specimens were control beams: the first one was designed to have deflection exceeding the code-permitted limits, whereas the second one was designed for code-compliant deflection limits. The other two specimens, which are similar to the first control beam, were retrofitted using CFRP sheets alone versus CFRP sheets combined with CFRP/honeycomb panels. This study contributes novel insights into the serviceability-focused retrofitting of RC wide beams, providing a framework for controlling excessive deflections through conventional CFRP and advanced CFRP-hybrid composite systems.

## 2. Experimental Campaign

### 2.1. Specimen Details

**Figure 2:**
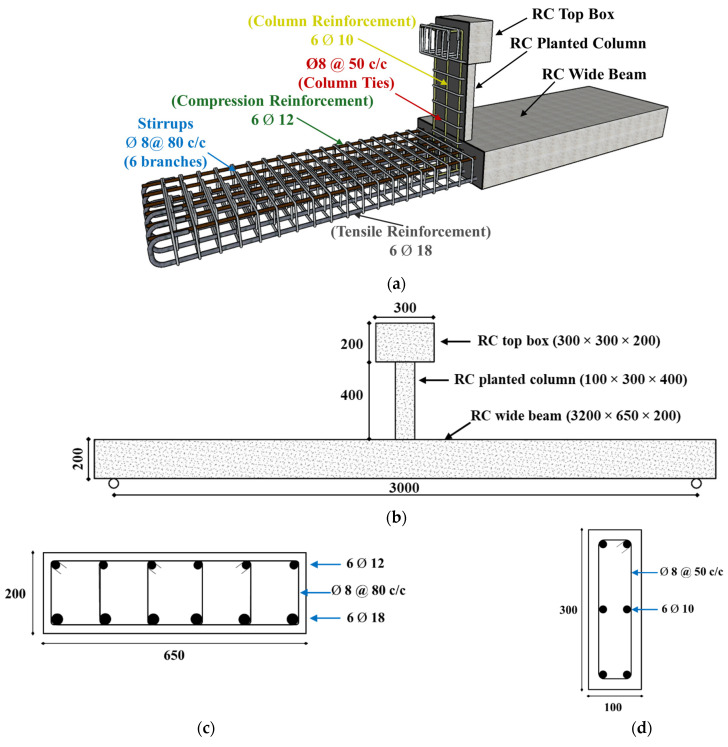
Details of control specimen WB-CON: (**a**) Steel reinforcement. (**b**) Concrete dimensions. (**c**) Beam section. (**d**) Column section (units: mm).

The testing campaign consists of testing four half-scale concrete wide beams carrying planted columns. The test matrix details are shown in Table 1. WB-CON which is the first control specimen is illustrated in Figure 2 along with its dimensions and reinforcement details. Specimen WB-CON had a wide beam measuring 650 mm in width, 200 mm in depth, and 3200 mm in total length, corresponding to a clear span of 3000 mm. The selected half-scale dimensions of test specimens were chosen to represent RC wide beams commonly used in Middle Eastern residential floors, where full-scale beams typically measure 1200–1400 mm in width and 300–500 mm in depth for spans of 6 to 8 m. The adopted scaling ensured that the experimental results remain representative of actual construction practice while being feasible for laboratory testing.

The specimen WB-CON was constructed with an RC planted column at its mid-span (Figure 2). The RC planted column has a rectangular section of 100 mm by 300 mm. It had an RC top box of dimensions 300 mm × 300 mm × 200 mm, as seen in Figure 2. The RC wide beam was reinforced with 6ϕ18 mm bars in tension and 6ϕ12 mm bars in compression, along with 6-leg stirrups of ϕ8 mm provided at 80 mm centers. The planted column was longitudinally reinforced with 6ϕ10 mm steel bars, along with ties of ϕ8 mm provided at 50 mm centers. As presented in Figure 2, the top box was heavily reinforced to avoid local failure during testing. WB-CON was designed as a half-scale representation of the concrete wide beams found in practice. As shown in Table 1 and Table 2, it satisfied the flexural and shear requirements of design codes [45,46] but exceeded the allowable deflection limit.

The chosen scale of test specimens balances practical limitations of the testing facility with the need to preserve stress distribution, stiffness ratios, and reinforcement anchorage conditions. Scale factors for reinforcement detailing and boundary conditions were maintained to achieve structural similitude in terms of stress transfer and deformation response.

Table 2 presents the calculated total deflections of the test specimens in comparison with the allowable deflections specified in the design codes [45,46]. The total deflection in Table 2 represents the sum of the instantaneous live-load deflection and the long-term deflection due to dead load and sustained live load. The sustained live load was assumed to be 25% of the total live load used in the design of residential buildings in Saudi Arabia [46]. The deflection values presented in Table 2 were computed using the detailed analytical procedure described in Section 4. The instantaneous deflection was calculated using standard elastic deflection equations with the effective moment of inertia, defined as a weighted average of the uncracked and fully cracked section properties. Long-term deflection was obtained by adding to this initial value an additional time-dependent component arising from creep and shrinkage under sustained loading. In accordance with ACI 318–19 [45], this component was evaluated by applying a time-dependent factor (λ) to the short-term deflection due to sustained loads, with the factor determined by the load duration and the amount of compression reinforcement.

**Figure 3:**
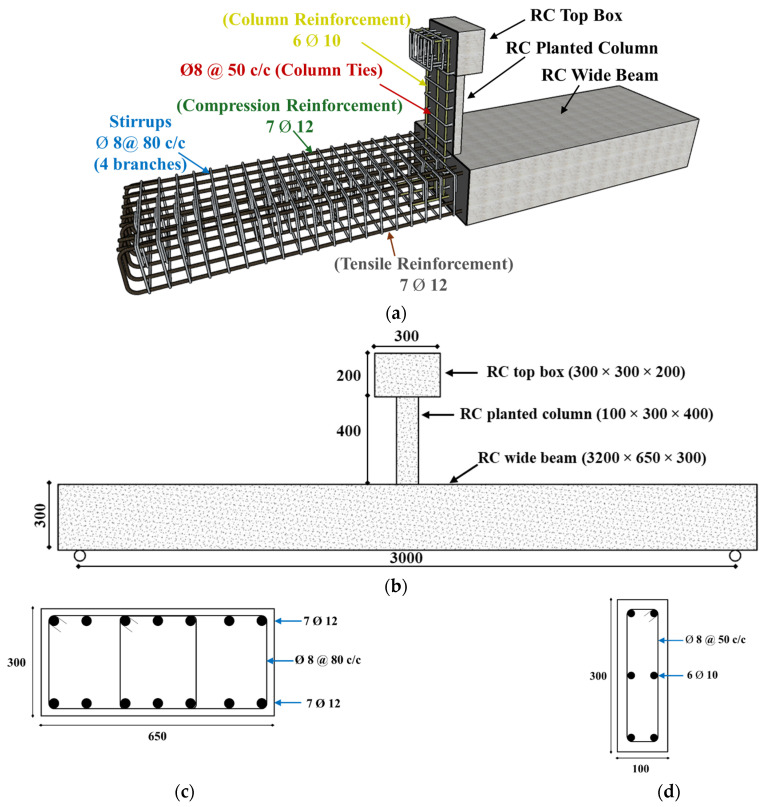
Details of control specimen WB-ACI: (**a**) Steel reinforcement. (**b**) Concrete dimensions. (**c**) Beam section. (**d**) Column section (unit: mm).

Another reference beam, WB-ACI, was designed to exhibit roughly the same bending strength as that of WB-CON, but with enhanced stiffness to satisfy code-specified deflection limits (see Table 2). Figure 3 depicts the details of the code-compliant beam WB-ACI. As seen in Figure 3, the second control beam, WB-ACI, had the same details as WB-CON except that the depth of the beam was increased to 300 mm, longitudinal beam reinforcement was 7ϕ12 mm (top and bottom), and the number of branches of beam stirrups was reduced to 4.

Thus, the two control specimens considered above both satisfy the code requirements for flexure and shear. However, only WB-ACI (Figure 3) satisfied the code deflection limit, whereas WB-CON (Figure 2) did not. Consequently, WB-ACI was constructed with a thickness of 300 mm and additional reinforcement, while WB-CON was limited to 200 mm thickness.

**Figure 4:**
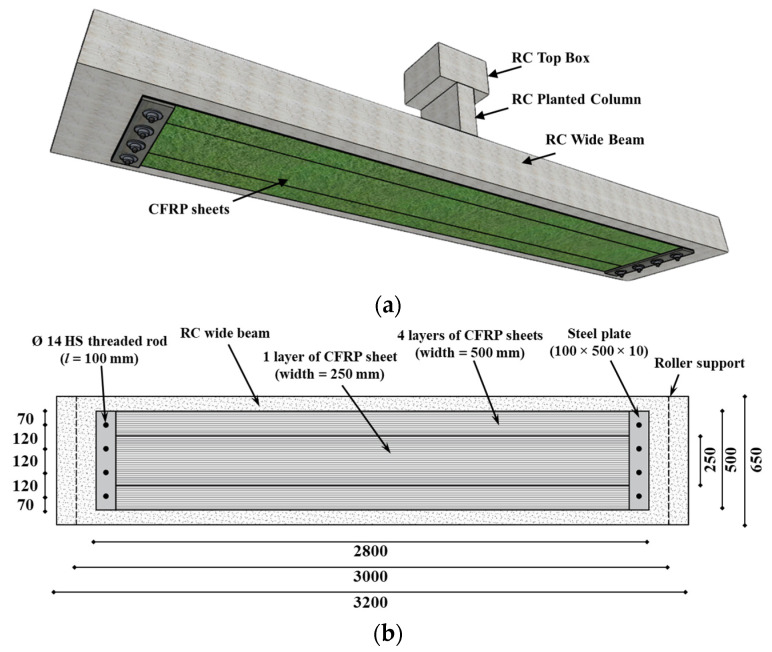
Details of strengthened specimen WB-CON-S1: (**a**) Isometric view. (**b**) Bottom view (unit: mm).

The last two specimens in the test matrix are the strengthened beams WB-CON-S1 and WB-CON-S2. These two specimens are identical to the first control specimen, WB-CON, in geometry and reinforcement. The strengthening schemes in the two upgraded beams were designed to enhance their stiffness to a level close to the second reference beam WB-ACI. Details of the strengthening scheme used in WB-CON-S1 are given in Figure 4. The beam was flexurally strengthened on the tension side using externally bonded CFRP sheets. This involved bonding of four CFRP layers of 500 mm width in addition to one CFRP layer of 250 mm width, as seen in Figure 4. To alleviate the unwanted end debonding of CFRP laminates, they were mechanically anchored at their ends using 10 mm thick steel plates of 50 × 100 mm size combined with 4 ϕ14 mm high-strength steel rods of 100 mm in length (see Figure 4).

**Figure 5:**
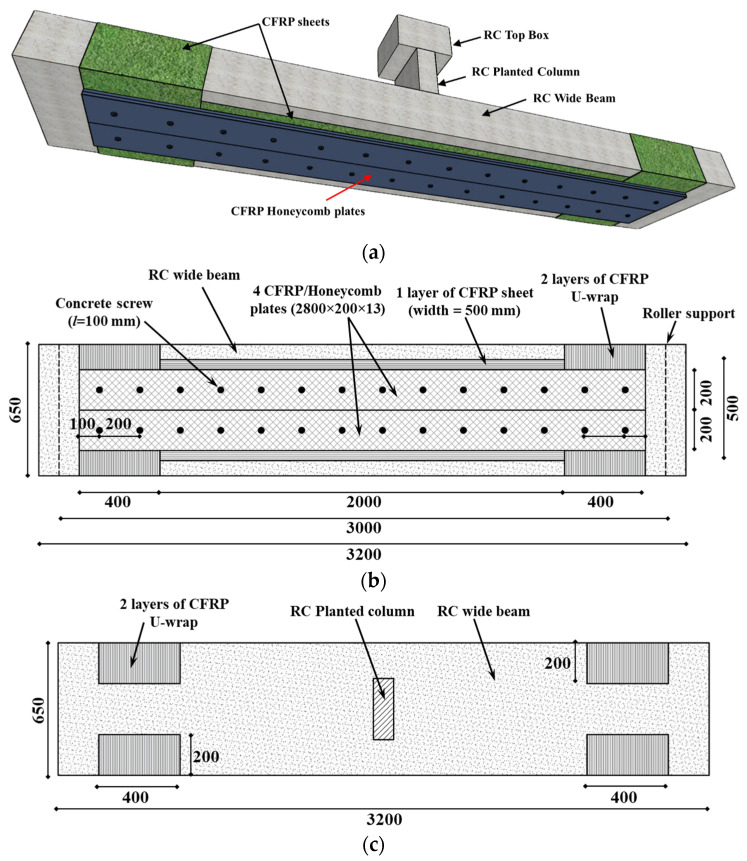
Details of strengthened specimen WB-CON-S2: (**a**) Isometric view. (**b**) Bottom view. (**c**) Top view (unit: mm).

Figure 5 illustrates the second strengthening scheme of the specimen WB-CON-S2. The beam was upgraded on the tension side using a hybrid system comprising one CFRP laminate of 2800 mm length by 500 mm width and four prefabricated CFRP/honeycomb sandwich plates of dimensions 2800 (length) × 200 (width) × 13 mm (thickness). The CFRP layer was attached to the tension side, and to alleviate the unwanted end debonding two U-wraps of CFRP (width = 400 mm) were installed at the CFRP sheet ends (see Figure 5). The four CFRP/honeycomb plates were not only adhesively bonded to the CFRP layer, but they were also mechanically installed to the bottom side with ϕ8 mm steel screw bolts (length = 100 mm) at an equal interval of 200 mm.

### 2.2. Properties of Materials

Compression tests were conducted as per ASTM testing standards [47] to characterize the mechanical properties of the concrete. Three concrete cylindrical specimens (150 mm × 300 mm) were cast concurrently with the test beams and cured under identical exposure conditions. Concrete’s compressive strength was 40 MPa on the testing day of the beams. The selected concrete grade represents the concrete commonly used in practice for similar structural elements. Additionally, the mechanical properties of the steel reinforcement used for both longitudinal and transverse reinforcement were obtained through standard tensile testing [48] and are reported in Table 3.

**Figure 6:**
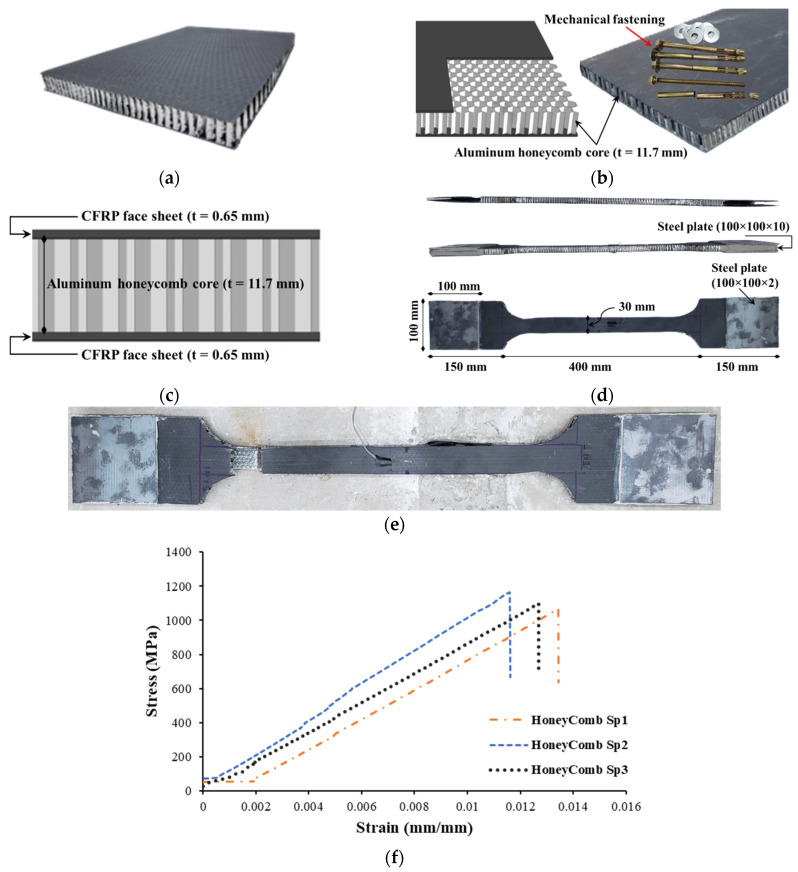
Dimensions and tensile coupons of CFRP/honeycomb plate: (**a**) CFRP/honeycomb panel. (**b**) Honeycomb core and mechanical anchoring system. (**c**) Section of CFRP/honeycomb plate. (**d**) Dimensions of CFRP/honeycomb testing coupon. (**e**) Failure pattern of testing coupon. (**f**) Stress–strain curve of tensile coupons.

Table 4 presents key information regarding the strengthening materials utilized in the experimental program. Specifically, the CFRP sheets are unidirectional and composed of high-strength carbon fibers. These sheets were attached using epoxy to the underside of beams with the help of an adhesive. The other strengthening material used in this investigation was the prefabricated CFRP/honeycomb panels. Carbon fiber/honeycomb sandwich panels are well-suited for various uses, fulfilling requirements for flatness, lightness, and rigidity. These panels consist of an aluminum honeycomb core sandwiched between self-adhesive layers of CFRP laminate skins. By incorporating an aluminum honeycomb core between carbon fiber skins, these sandwich panels provide enhanced strength compared to the skins individually. In addition, the inclusion of a core material in this system offers an additional mechanism for enhancing the energy absorbed. Furthermore, the panel length is adaptable, which can be conveniently cut to the required dimensions on-site using standard woodworking tools. The total thickness of the CFRP/honeycomb was 13 mm (face sheet laminate = 0.65 mm, aluminum core = 11.7 mm). Concrete screws of 8 mm diameter were used to mechanically fasten the honeycomb panels to the concrete, spaced at 200 mm centers (refer to Figure 5). The use of mechanical fastenings in securing the sandwich panels also facilitates the extrusion of extra epoxy, thereby ensuring full bond development between the panel and the strengthened specimen. Standard tensile test coupons were prepared for each strengthening material and the tests were conducted as per the ASTM standard [48,49]. CFRP/honeycomb coupons were prepared and fabricated from the composite system (see Figure 6). As illustrated in the figure, the honeycomb core material was removed at the ends subjected to gripping to avoid crushing of the relatively soft core. The resulting voids between the face sheets were filled with rectangular steel plates matching the thickness of the core material. Table 4 summarizes the test results, and the corresponding stress–strain response is presented in Figure 6.

It should be noted that bond durability and creep under sustained loading were not investigated for the strengthening materials in the present study. The epoxy adhesive properties, reported in Table 4, were obtained from the manufacturer’s datasheets, and they reflect the short-term behavior. Caution should be exercised when extrapolating to long-term or environmentally exposed conditions.

### 2.3. Preparation of Test Specimens

Reinforcing steel bars were prepared and inspected in accordance with the design specifications. Following this, the formwork was assembled, and strain gauges were affixed on selected reinforcement bars as specified in the instrumentation layout for test measurements. Final quality assurance checks were then conducted to confirm readiness for casting. Ready-mix concrete was supplied by a local producer, and all specimens were cast concurrently to eliminate variability between concrete batches. After curing of the wide beam with planted column specimens, the beams were prepared for strengthening. For this purpose, the beam surfaces were cleaned and sandblasted to remove all unevenness from the surfaces. After that, the specimens were ready for the strengthening process.

**Figure 7:**
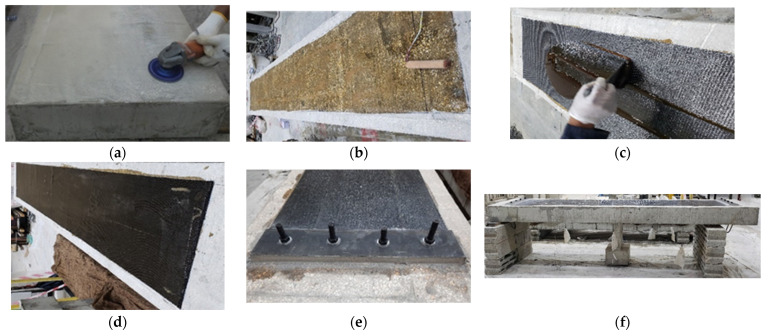
Strengthening process of specimen WB-CON-S1: (**a**) Surface treatment and cleaning. (**b**) Application of epoxy coating on concrete. (**c**) Bonding of 1st CFRP layer. (**d**) Completion of CFRP strengthening. (**e**) End anchorage using bolted steel plates. (**f**) Specimen ready for testing.

For WB-CON-S1, a thin layer of adhesive was spread on the prepared beam’s bottom surface. First, the CFRP sheet was positioned on the beam’s bottom surface, ensuring the removal of all air voids to achieve full contact. Subsequently, a second resin layer was applied over the first sheet to achieve full saturation and to prepare the surface for the application of the next layer. This process was repeated until five CFRP sheets were installed. Upon completion, a plastic cover was placed over the bonded layers to protect them during the resin curing process. Following curing, 10 mm thick steel plates of 500 × 100 mm size were bonded and mechanically anchored at the ends of the CFRP laminates using SIKA 31/41 adhesive and four high-strength threaded rods. Figure 7 illustrates the strengthening procedure for the specimen WB-CON-S1.

**Figure 8:**
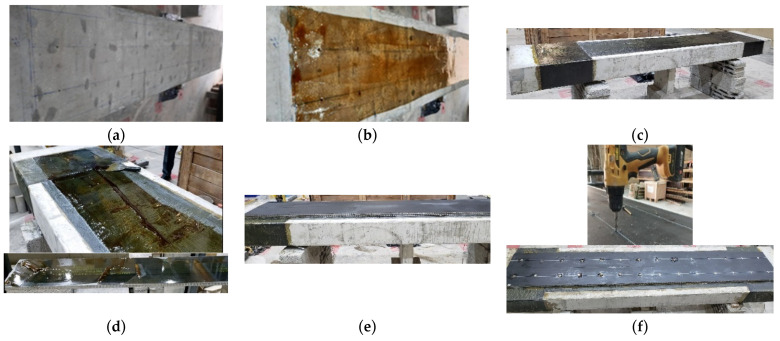
Strengthening process of specimen WB-CON-S2: (**a**) Surface treatment and cleaning. (**b**) Application of epoxy coating on concrete. (**c**) Bonding of CFRP layer. (**d**) Application of epoxy on CFRP sheet and CFRP/honeycomb plates. (**e**) Bonding of CFRP/honeycomb plates. (**f**) Application of mechanical anchorage for CFRP/honeycomb plates.

For WB-CON-S2, the bonding positions of the CFRP sheets and concrete screws were first marked on the beam. A drilling machine was then used to create pilot holes for the screws. These holes were sized to accommodate 8 mm diameter concrete screws, which were installed at uniform intervals of 200 mm to mechanically secure the honeycomb panels in place. Then, the standard manufacturer’s recommended procedures were adopted to bond the CFRP sheet. Then, two U-wrapped CFRP sheets were applied as anchorage for the CFRP layer. After the curing period for the epoxy resin, the honeycomb panels were installed. Two layers of CFRP/honeycomb panels were used; each layer had two panels of 2800 × 200 × 13 mm each. The CFRP sheet and the CFRP/honeycomb panels were coated with epoxy adhesive. The panels were then bonded to the CFRP sheet along the beam’s bottom surface. Another layer of epoxy was subsequently applied to the exposed surface of the CFRP/honeycomb panels to facilitate the installation of an additional panel layer. Following this, the panels were cored at the locations of the pre-drilled pilot holes, and the concrete screws were inserted and tightened. To ensure optimal bond quality and uniform adhesion, clamps were used to apply pressure on the sandwich panels. Figure 8 illustrates the strengthening procedure for the specimen WB-CON-S2.

The CFRP sheets were applied following the wet lay-up procedure. Concrete surfaces were mechanically roughened and cleaned to remove laitance and contaminants, after which a primer coat was applied (with consumption rate of 0.3–0.5 kg/m^2^) to enhance adhesion. The two-component epoxy adhesive was mixed in controlled proportions and applied as a uniform film, followed by placement and impregnation of the CFRP sheets using rollers to ensure full saturation and elimination of voids. For multi-ply systems, successive layers were applied in sequence with proper consolidation. The epoxy consumption rate was 0.5–0.8 kg/m^2^ per layer. Curing was carried out under ambient laboratory conditions for 7 days. Quality control comprised systematic visual inspection for wrinkles, voids, and incomplete saturation.

Although the aluminum core was fully isolated from contact with other potentially galvanically active materials, the only interface with steel occurs at the bolt shank, which is recommended to be epoxy-coated to prevent galvanic corrosion. As a result, galvanic corrosion is not anticipated in the adopted scheme. While environmental degradation and long-term creep of the CFRP-honeycomb system are relevant for service-life assessment, these effects were beyond the scope of the present short-term experimental program. These considerations are noted here to clarify the applicability and limitations of the results.

### 2.4. Instrumentation and Testing

**Figure 9:**
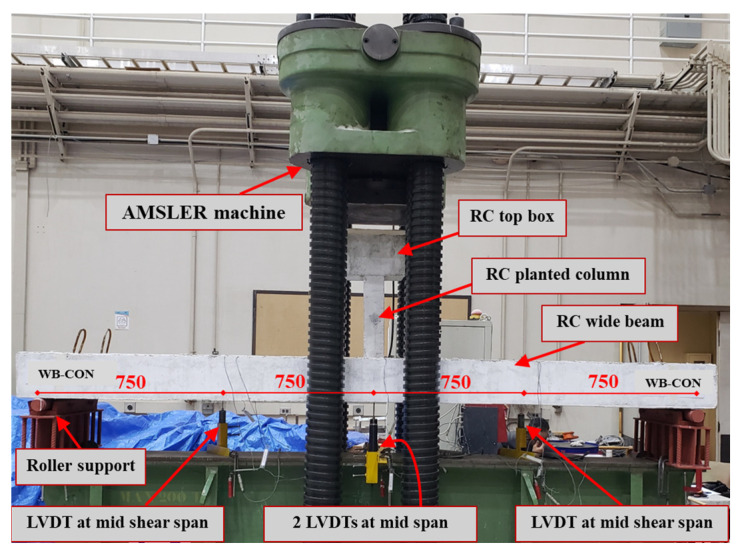
Test setup and instrumentation details (unit: mm).

The beams were tested with the help of the AMSLER test machine (ZwickRoell, Switzerland) of 2000 kN total capacity, as illustrated in Figure 9. The tests were conducted by increasing deflection at 2 mm/min. A load cell, integrated into the testing machine, was used to record the applied load, which was exerted directly through the planted column. The clear span of simply supported beams was 3000 mm, with shear spans of 1500 mm on each side. Figure 9 also depicts the experimental setup and instrumentation layout. The deflection of the beam’s mid-span was recorded using 2 Linear Variable Displacement Transducers (LVDTs), with additional LVDTs positioned in each shear span. Furthermore, strain gauges were installed to monitor strain responses in the longitudinal and transverse rebars, CFRP sheets, and CFRP/honeycomb panels. The tests were conducted under monotonic loading; however, the loading was staged with brief pauses at selected load levels to allow for the marking of cracks and recording of observations. The monotonic loading was considered to focus on the fundamental load–displacement response, cracking patterns, and ultimate strength of the specimens under controlled conditions. Monotonic loading enabled precise measurement and observation of these behaviors without the additional complexity introduced by sustained or cyclic effects. While long-term sustained loads and cyclic/seismic loadings are relevant for serviceability and seismic performance, they were beyond the scope of the present study. However, future work is recommended to investigate the performance of similar specimens under time-dependent and cyclic/seismic loading conditions.

## 3. Experimental Results and Discussion

**Figure 10:**
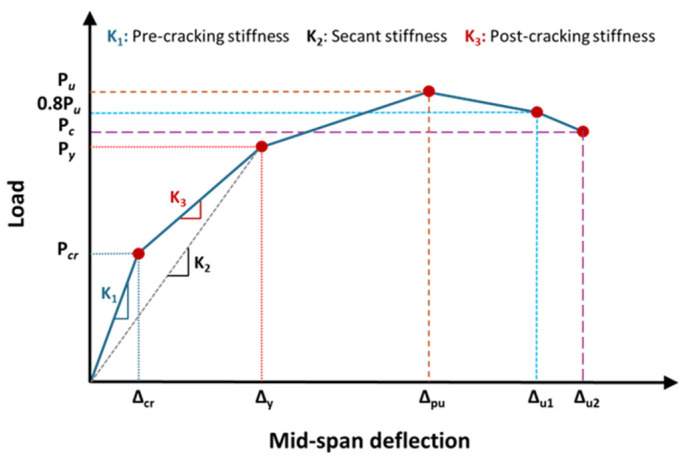
Definition of key parameters of load–deflection plot for test specimens.

Table 5 illustrates the key experimental results of tested beams, including the first crack load (P_cr_) and associated deflection (Δ_cr_), the load at the initiation of yielding (P_y_) of the main tension reinforcement along with its associated deflection (Δ_y_), the peak load (P_u_) and corresponding deflection (Δ_pu_), the deflection at the ultimate state (Δ_u1_), and the observed failure mode. The ultimate state was assumed as the post peak point at which load is reduced by 20% (0.8P_u_), as illustrated in Figure 10, where P_c_ and Δ_u2_ are the load at concrete crushing and its associated deflection.

### 3.1. Failure Modes and Flexural Capacity

**Figure 11:**
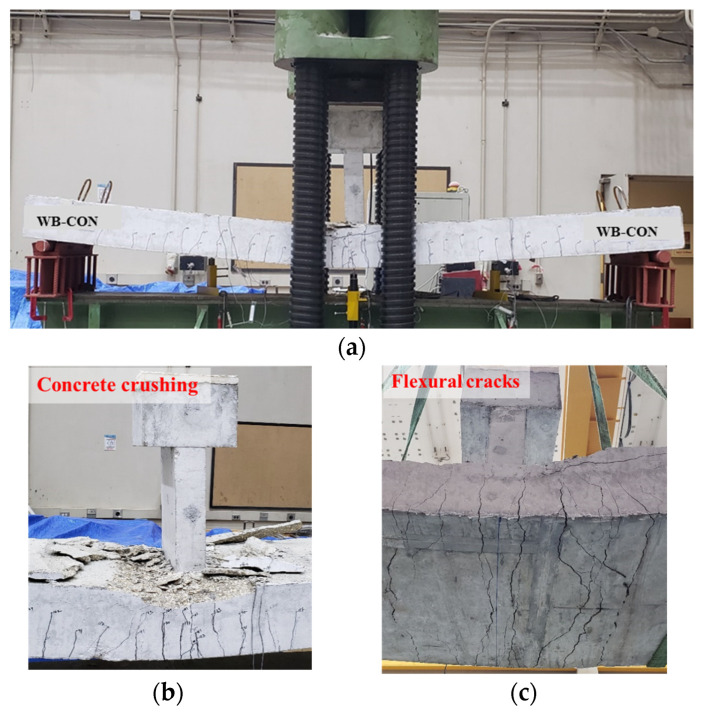
Failure pattern of control beam WB-CON: (**a**) Final deflected shape. (**b**) Close-up picture showing concrete crushing. (**c**) Bottom concrete surface showing flexural cracking.

**Figure 12:**
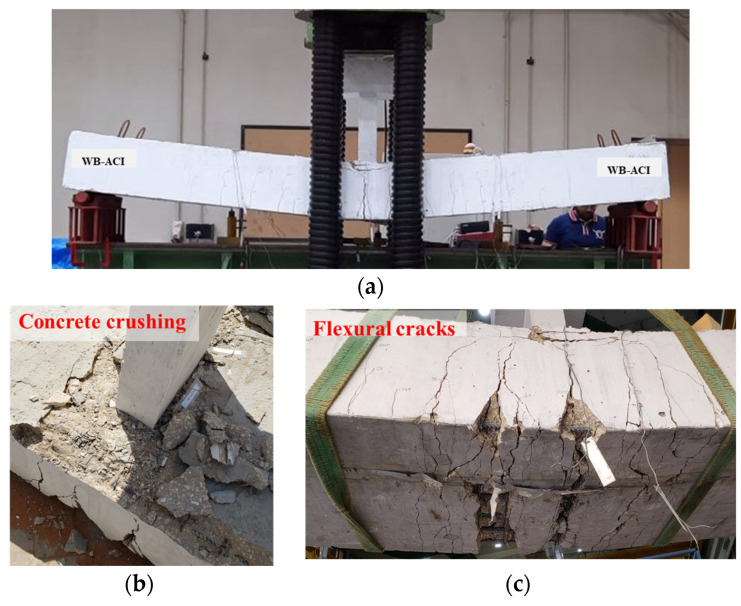
Failure pattern of control beam WB-ACI: (**a**) Final deflected shape. (**b**) Close-up picture showing concrete crushing. (**c**) Bottom concrete surface showing flexural cracking.

Figure 11 and Figure 12 illustrate the failure modes of the control specimens WB-CON and WB-ACI. Both beams exhibited the same flexural failure mode involving major flexure cracks at the beam’s mid-span. The flexural cracks appeared after yielding of the longitudinal rebars and progressed nearly vertically toward the beam’s top surface. As the deflections increased, the concrete in the compression region was crushed. The peak loads of both specimens were nearly identical, as the beams were designed to possess equivalent flexure capacities.

**Figure 13:**
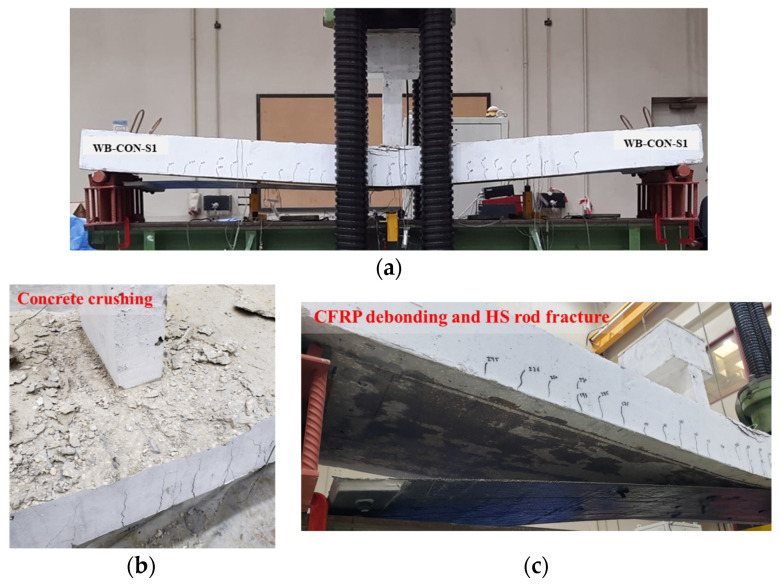
Failure pattern of retrofitted specimen WB-CON-S1: (**a**) Final deflected shape. (**b**) Close-up picture showing concrete crushing. (**c**) Bottom surface showing CFRP debonding.

**Figure 14:**
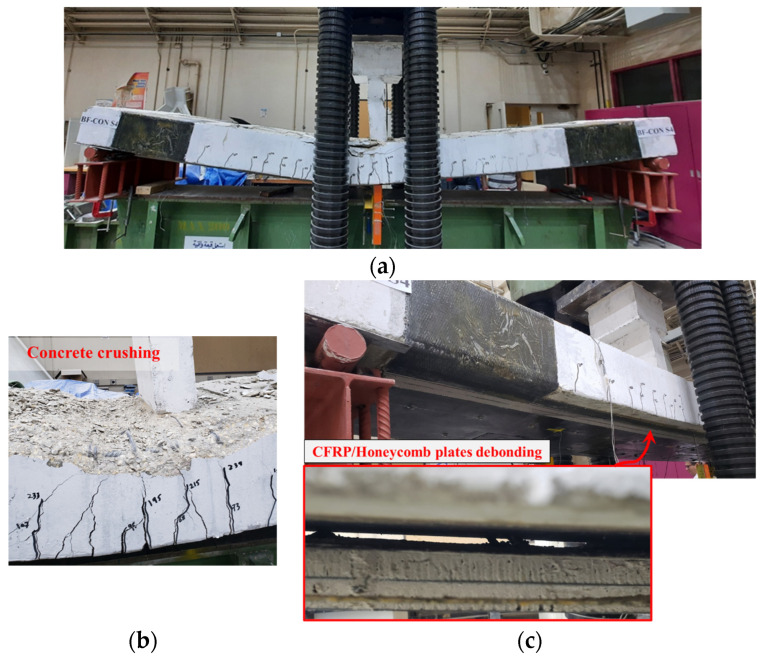
Failure pattern of retrofitted specimen WB-CON-S2: (**a**) Final deflected shape. (**b**) Close-up picture showing concrete crushing. (**c**) Bottom surface showing CFRP/honeycomb debonding.

Figure 13 and Figure 14 depict the failure modes of the strengthened beams WB-CON-S1 and WB-CON-S2, respectively. The specimen WB-CON-S1 exhibited intermediate crack (IC) debonding of the CFRP laminate, which started at mid-span and propagated toward the support following the tension rebars’ yielding. This was subsequently accompanied by concrete crushing in the compression region near the planted column. In the case of WB-CON-S2, failure occurred through debonding of the CFRP/honeycomb panel and crushing of concrete in the compression region around the planted column after yielding of the tensile steel. The use of CFRP sheets alone and the hybrid system of CFRP sheets combined with CFRP/honeycomb panels proved effective in enhancing the beams’ ultimate capacity. Specifically, the ultimate capacity of WB-CON-S1 increased by 65% in comparison to the unretrofitted beam because of the presence of CFRP reinforcement, while WB-CON-S2 demonstrated a 71% increase attributed to the combined use of CFRP sheets and CFRP/honeycomb panels.

For WB-CON-S1, the use of end anchorage of CFRP sheets with steel plates effectively delayed premature end pull-off but was limited in confining intermediate cracks along the span. In contrast, the U-wraps and bolts applied in WB-CON-S2 provided more continuous restraint against peeling and separation, which contributed to improved load transfer and delayed IC debonding, thereby enhancing overall performance.

The flexural capacity enhancements achieved in this study align well with findings reported for wide shallow beams subjected to different loading conditions [1,37]. While the loading configuration adopted here—a concentrated load applied through a planted column—induces a more severe stress concentration compared to the four-point bending tests conducted by Al-Negheimish et al. [1] and El-Sayed et al. [37], the observed ultimate capacity increases of 65–71% in the strengthened beams are comparable to the 70–77% increases they reported. Moreover, intermediate crack (IC)-induced debonding following steel yielding remained the predominant failure mode, even under the intensified stress concentrations generated by the planted column.

The observed failure mode carries important practical implications, underscoring a key strength of this study’s findings. Debonding was initiated only after substantial steel yielding, thereby providing a ductile warning mechanism and demonstrating the system’s reliable performance under ultimate loads. In addition, the use of robust mechanical anchorages effectively addressed long-term durability concerns, mitigating the inherent limitations of adhesive-only bonding. Collectively, these outcomes validate a retrofitting strategy that ensures both dependable structural performance and enhanced durability in practical applications.

### 3.2. Load–Displacement Response

**Figure 15:**
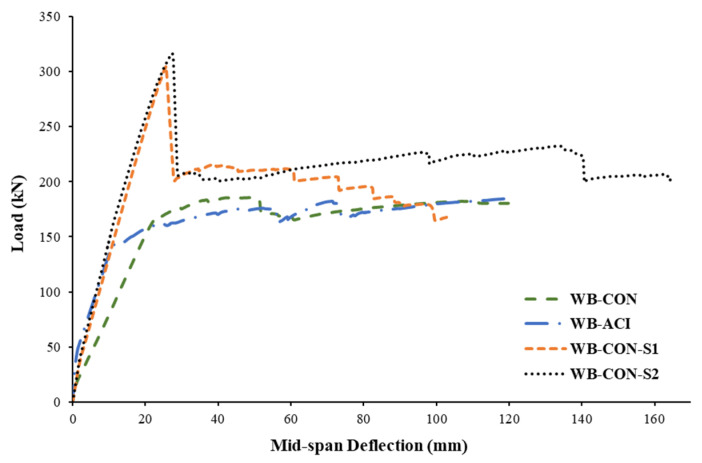
Load–deflection plots for tested beams.

Figure 15 presents the load–deflection responses of the tested specimens. Both control specimens exhibited typical behavior associated with tension-controlled reinforced concrete (RC) beams, characterized by a trilinear load–deflection relationship as depicted in Figure 10 and Figure 15. The initial linear segment corresponds to the uncracked stage, marked by relatively high stiffness, where the entire uncracked concrete section and steel reinforcement work together to resist the load. Thus, the stiffness is at its maximum, defined by the beam’s gross moment of inertia. Following the onset of concrete cracking, the first significant drop in stiffness occurs upon the initiation of flexural cracks in the concrete’s tension zone. At this point, the tensile stresses are transferred to the steel reinforcement and the externally bonded strengthening system. The stiffness is now dictated by the cracked transformed section’s moment of inertia, resulting in a reduced slope on the load–deflection curve. The third segment of the load–deflection response began when the second major stiffness degradation occurred, when the main tensile steel reinforcement yielded. Beyond this point, the beam’s behavior is characterized by a yielding plateau, where large deflections occur with small increments in load. The resistance is primarily provided by the strain hardening of the steel and the linear–elastic behavior of the CFRP reinforcement, which continues to carry increasing tensile stress until the ultimate failure of the system. The control specimen, WB-ACI, demonstrated substantially higher stiffness throughout the test in comparison to WB-CON. This enhanced performance is attributed to the design of WB-ACI in compliance with current code-specified deflection limits [45,46]. Both strengthened beams (WB-CON-S1 and WB-CON-S2) exhibited higher stiffness in comparison to the control beam WB-CON and approximately the same stiffness as the control specimen WB-ACI. As seen from Figure 15, the strengthened WB-CON-S2 has a more yielding plateau compared to the strengthened beam WB-CON-S1. This is due to the introduction of CFRP/honeycomb plates, which added more ductility to the beam.

It is identified from Figure 15 and Table 5 that the strengthening systems considerably improved the load–deflection behavior of the beams WB-CON-S1 and WB-CON-S2 compared with the control beam WB-CON. In WB-CON-S1, the inclusion of CFRP sheets enhanced yield and peak loads by about 81% and 65%, respectively. Nevertheless, CFRP sheets reduced the deflection at peak load of WB-CON-S1 by about 48%. In the specimen WB-CON-S2, the addition of CFRP sheets and honeycomb plates enhanced the peak load by 71% and the yield load by 92%. This high gain in yield load reflects the efficiency of this hybrid system in mobilizing additional tensile resistance beyond what is typically achieved with CFRP sheets alone. However, it reduced the deflection at peak load by 45%. When compared with the control wide beam WB-ACI, the strengthening systems increased the yield load by 85% and 96% for WB-CON-S1 and WB-CON-S2, respectively. However, the improvements in the ultimate load owing to strengthening were almost similar to those compared with the reference beam WB-CON.

Table 6 depicts the experimental values of stiffnesses for pre-cracking, secant, and post-cracking stages. Additionally, the dissipated energies and displacement ductility of the tested beams are also reported in the table. These evaluation parameters are calculated as indicated in the schematic diagram in Figure 10. The post-cracking stiffness was estimated as the gradient of the line joining the onset of cracking and yielding on the load–displacement curve. The secant stiffness was estimated as the ratio of the yielding load to the corresponding deflection. Pre-cracking stiffness was estimated as the ratio of the cracking load to the corresponding deflection. The energy dissipated by the beam is represented by the area beneath the load–displacement curve up to the crushing of concrete. Ductility was evaluated as the ratio of ultimate to yield deflection. It can be noted that, for the strengthened beam WB-CON-S1, its flexural stiffness was enhanced by 63% in comparison with the control specimen WB-CON, which is nearly equivalent to that of the control specimen WB-ACI, only differing by 6%. Furthermore, the test results indicated a 3% enhancement in dissipated energy compared to the unstrengthened specimen WB-CON. However, it decreased by 1% compared with the control specimen WB-ACI. Additionally, the strengthened beam WB-CON-S1 exhibited a reduction in ductility by 23% and 57% compared to the two control specimens WB-CON and WB-ACI, respectively. For the strengthened beam WB-CON-S2, its flexural stiffness was enhanced by 67% as compared to the control specimen WB-CON. The stiffness of the strengthened specimen WB-CON-S2 was nearly comparable to that of the ideal control specimen WB-ACI, with only a 4% difference. Moreover, the experimental results demonstrated a substantial increase in dissipated energy, reaching 80% and 74% higher than that of the control specimens WB-CON and WB-ACI, respectively. Additionally, the displacement ductility of WB-CON-S2 increased by 20% compared to WB-CON, but was 33% lower than that of WB-ACI. Although the strengthened specimens exhibited lower ductility values, these values remain within acceptable limits for RC members. The reduction in ductility is mainly due to the increased stiffness of the strengthening system. However, in terms of serviceability, the improved stiffness and reduced deflection enhance performance under service loads.

The significant improvement in flexural stiffness for the retrofitted beams is a key finding that aligns with previous research. Al-Negheimish et al. [1] similarly found that all their strengthened wide beams exhibited much stiffer behavior than the unstrengthened control beam. They also noted that the degree of stiffness enhancement was directly proportional to the axial stiffness of the CFRP reinforcement, a principle that is also reflected in our results. However, this gain in stiffness often comes at the cost of ductility. The 23% reduction in displacement ductility for our CFRP-only specimen (WB-CON-S1) is a well-documented phenomenon. Al-Negheimish et al. [1] also reported a significant loss in ductility, with the strengthened beams retaining only 42–49% of the control beam’s deflection ductility and exhibiting no post-yield plateau.

### 3.3. Load–Strain Response

**Figure 16:**
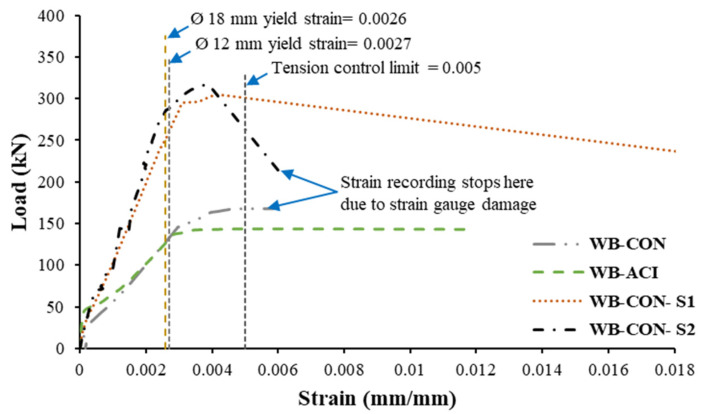
Load versus strain in tension steel rebars of test specimens at mid-span.

**Figure 17:**
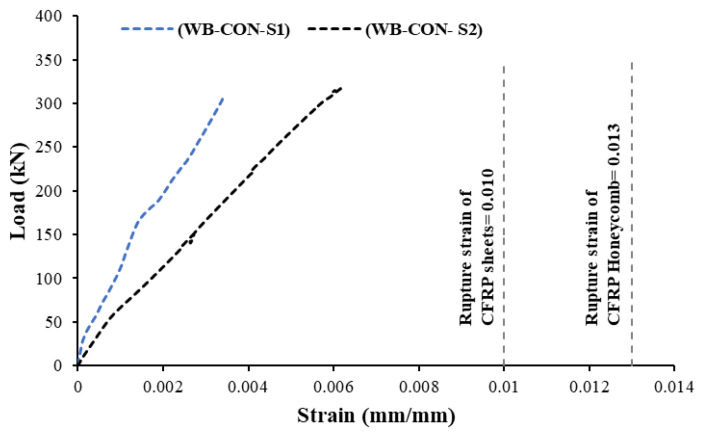
Load versus strain in strengthening layers of strengthened specimens at mid-span.

Figure 16 depicts the variation in rebar strains at mid-span against applied load for all tested specimens. As evident from the figure, all specimens met the tension-controlled behavior criteria specified in the current codes [45,46], as the maximum rebar strains are substantially greater than 0.5%. Additionally, the tensile reinforcement yielded prior to reaching the ultimate load in each case. Figure 17 presents the variation in strain at mid-span against load for CFRP sheets and CFRP/honeycomb plates for the strengthened wide beams WB-CON-S1 and WB-CON-S2, respectively. The peak strain in the CFRP sheet of the specimen WB-CON-S1 was significantly lower than its ultimate rupture strain. This is because the rupture of the CFRP sheet did not occur; instead, failure was governed by premature debonding, as previously discussed. Similarly, the peak strain recorded in the CFRP/honeycomb panels of the specimen WB-CON-S2 was markedly lower than the corresponding rupture limit. This is because of the premature debonding of the CFRP/honeycomb panels, which occurred before the panels reached their ultimate strain capacity.

### 3.4. Comparison of Strengthening Schemes

**Figure 18:**
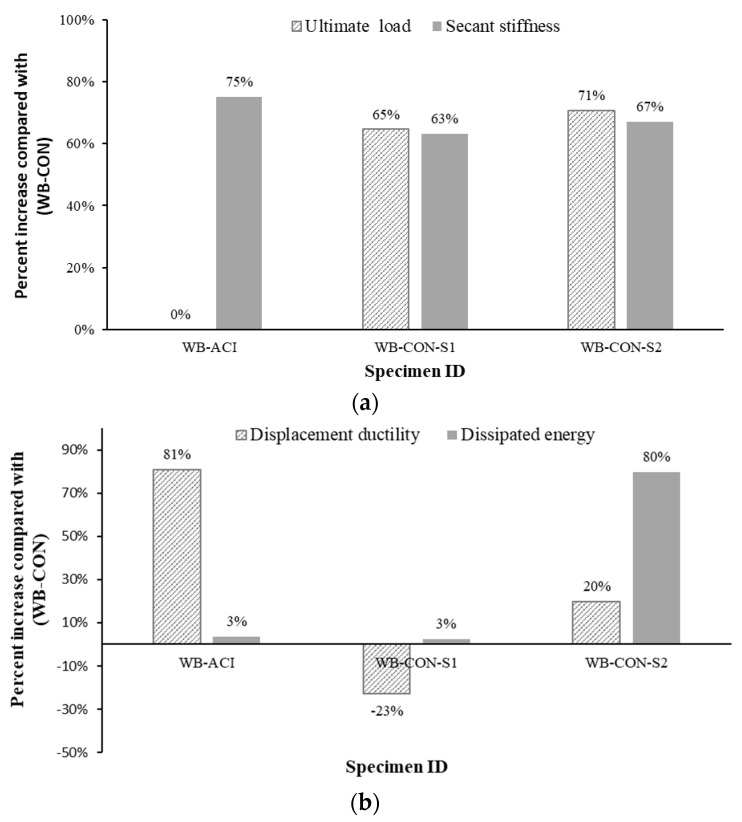
Percent increase compared with control specimen WB-CON: (**a**) Ultimate load and secant stiffness. (**b**) Displacement ductility and dissipated energy.

Both strengthened specimens, WB-CON-S1 and WB-CON-S2, demonstrated enhanced flexural performance in comparison to the control wide beam WB-CON. As illustrated in Figure 15, both strengthened specimens demonstrated higher load and stiffness, as a result of introducing the strengthening materials of CFRP sheets and CFRP/honeycomb plates. While both retrofitting techniques proved highly effective, a deeper comparison reveals critical differences in their structural behavior and failure mechanisms. Both WB-CON-S1 and the hybrid system WB-CON-S2 provided significant and comparable increases in flexural strength and stiffness. The secant stiffness for WB-CON-S1 and WB-CON-S2 increased by 63% and 67%, respectively, relative to WB-CON, successfully controlling deflection to within serviceability limits. The ultimate failure mode for both was also similar: yielding of the main tension steel followed by intermediate crack (IC) debonding of the external reinforcement and subsequent concrete crushing. However, the systems diverged significantly in their post-yield behavior. The most notable advantage of the hybrid system was its superior energy dissipation capacity and ductility. The inclusion of CFRP/honeycomb plates in WB-CON-S2 led to an 80% increase in dissipated energy and a 20% increase in displacement ductility when compared to the unstrengthened WB-CON beam. In contrast, the CFRP-only system in WB-CON-S1, while increasing its strength, resulted in a 23% reduction in ductility. The mechanism behind this enhanced performance in WB-CON-S2 stems from the composite nature of the honeycomb sandwich panels. The aluminum honeycomb core is specifically designed to absorb energy through the plastic deformation and crushing of its thin cell walls under high stress. This provides an additional energy dissipation path that is not available in the linear–elastic CFRP sheet system. Furthermore, the use of mechanical screw-bolts in WB-CON-S2, in addition to adhesive bonding, likely created a more damage-tolerant connection that helped to arrest the propagation of debonding, allowing the beam to undergo larger deflections post-yield and thereby extending the yielding plateau seen in Figure 15. This combination of material properties and connection detail explains the superior and more ductile performance of the hybrid strengthening strategy. Figure 18 depicts the percent increase in the evaluated parameters of test specimens compared to the control specimen WB-CON.

The different anchorage systems used for the strengthened specimens likely influenced their post-yield behavior. Both the steel plate end-anchors (WB-CON-S1) and the combination of CFRP U-wraps and distributed screw anchors (WB-CON-S2) were highly effective in preventing premature end debonding, forcing failure to initiate in the high-moment mid-span region. However, the distributed nature of the screw anchors in the hybrid system likely contributed to its superior ductility and energy dissipation. These screws, installed at regular intervals, mechanically stitched the panels to the beam and controlled the propagation of the debonding, thereby helping to maintain composite action even after failure initiates. This controlled failure progression likely explains the more stable and extended yielding plateau observed in WB-CON-S2′s load–deflection curve compared to the more abrupt failure observed in WB-CON-S1 after debonding began.

From a practical standpoint, both strengthening schemes offer excellent constructability. The application of CFRP composites is a straightforward process, and the hybrid system (WB-CON-S2) further streamlines installation through the use of easy-to-handle prefabricated panels and standard mechanical fasteners. When compared to traditional strengthening methods, the advantages of both investigated systems are particularly compelling. Their lightweight nature makes installation significantly easier and safer than conventional steel plate bonding, and their noncorrosive properties enhance long-term durability. Furthermore, both techniques are minimally intrusive, adding negligible weight and preserving architectural clearances, unlike the disruptive and dimension-altering process of concrete jacketing. This positions these advanced composite systems as highly efficient and practical alternatives for deflection control in existing structures.

Although both strengthening schemes are practically feasible, the first scheme using CFRP sheets alone may be more slightly time-consuming due to the large number of layers and the use of mechanical anchors. In contrast, the hybrid CFRP–honeycomb scheme requires only a single CFRP layer, but the installation of the honeycomb plate necessitates fasteners and drilling. Therefore, the overall time and effort involved in both schemes are expected to be comparable.

While the study focuses on beams representative of Saudi residential construction, the proposed strengthening methods are not limited to this context. The strengthening techniques and associated detailing principles are applicable to a wide range of RC beams in different construction practices and climates, provided that material properties and environmental considerations are appropriately accounted for.

## 4. Analytical Study

**Figure 19:**
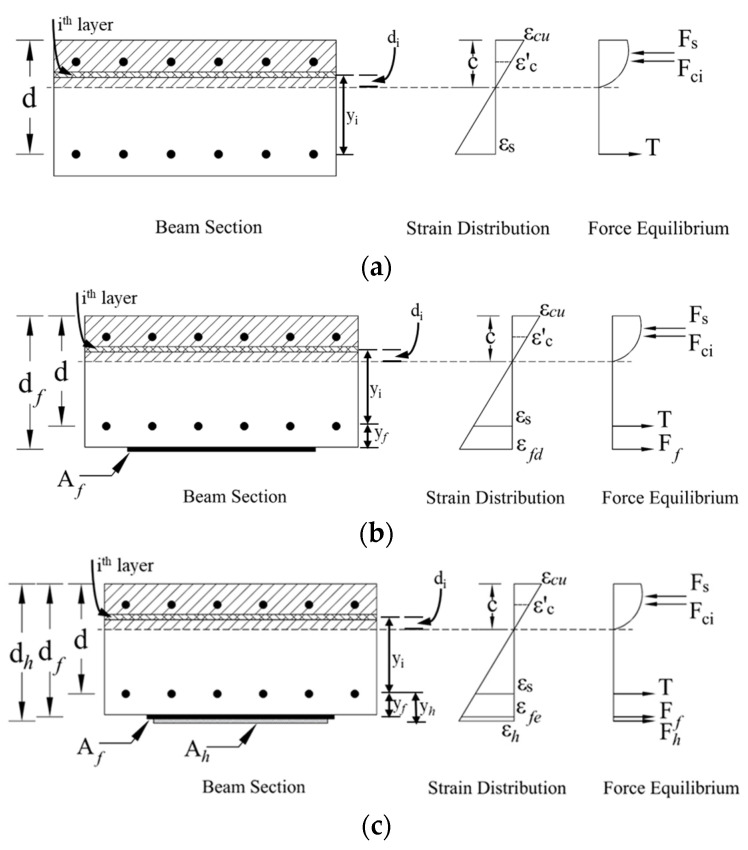
Strain and stress variation for (**a**) control specimens; (**b**) WB-CON-S1; (**c**) WB-CON-S2 (laminar analysis approach).

Using the laminar analysis approach, the cross-section of the wide beam was discretized into multiple concrete layers, as illustrated in Figure 19. For each layer, Mander’s model [50] was employed to estimate the concrete stress. The neutral axis depth was estimated through an iterative procedure, in accordance with current design standards [45,46,51], to satisfy the force equilibrium between the concrete and reinforcing steel in the control specimens WB-CON and WB-ACI. For the retrofitted wide beams, equilibrium was established among the concrete, steel reinforcement, and CFRP reinforcement. The tensile force of concrete was neglected in the analysis.

For strengthened beam WB-CON-S2, the force equilibrium equation is

(1) $$F_{s}+F_{ci}=T+F_{f}+F_{h}$$

where $F_{s}$ is the compression steel force, $F_{ci}$ is the compressive force in ith layer of concrete, *T* is the tension steel force, $F_{f}$ is the CFRP sheet force, and $F_{h}$ is the CFRP/honeycomb panel force. These forces can be calculated as

(2) $$F_{ci}=f_{ci}A_{i};\;F_{s}={f^{\prime }}_{s}{A^{\prime }}_{s};\;F_{f}=f_{f}A_{f};\;F_{h}=f_{h}A_{h};\;T=f_{s}A_{s}$$

where $f_{ci}$ and $A_{i}$ are the stress and area of the ith concrete layer; ${f^{\prime }}_{s}$ and ${A^{\prime }}_{s}$ represent the stress and area of rebars in compression; $f_{s}$ and $A_{s}$ are the stress and area of rebars in tension; $f_{f}$ and $A_{f}$ are the stress and area of CFRP reinforcement; and $f_{h}$ and $A_{h}$ are the stress and area of CFRP/honeycomb panels. The stress in each concrete layer was computed using Mander’s model [50], based on the strain values in the respective layers. For rebars in both tension and compression zones, a bilinear stress–strain model was considered to evaluate the corresponding stress values.

These strains are calculated to achieve the force equilibrium as follows.

(3) $$\varepsilon_{ci}=\varepsilon_{cu}\left(\frac{d_{ci}}{c}\right);\;{\varepsilon^{\prime }}_{s}=\varepsilon_{cu}\left(\frac{c-d^{\prime }}{c}\right);\;\varepsilon_{s}=\varepsilon_{cu}\left(\frac{d-c}{c}\right)$$

(4) $$\varepsilon_{fe}=\varepsilon_{cu}\left(\frac{d_{f}-c}{c}\right)\;\;\leq \varepsilon_{fd};\;\varepsilon_{he}=\varepsilon_{cu}\left(\frac{d_{h}-c}{c}\right)\;\;\leq \varepsilon_{fd}$$

(5) $$\varepsilon_{fd}=0.41\;\sqrt{\frac{{f^{\prime }}_{c}}{nE_{f}t_{f}}}$$

After validation of Equation (1), the flexural capacity was calculated as

(6) $$M_{n}=\sum_{i}^{n}F_{ci}Y_{i}+F_{s}\left(d-d^{\prime }\right)+F_{f}\left(d_{f}-d\right)+F_{h}\left(d_{h}-d\right)$$

where $Y_{i}$ denotes the distance between the tension reinforcement and the ith concrete layer.

Deflection calculations in this study followed the approach recommended by the ACI [45,52].

**Figure 20:**
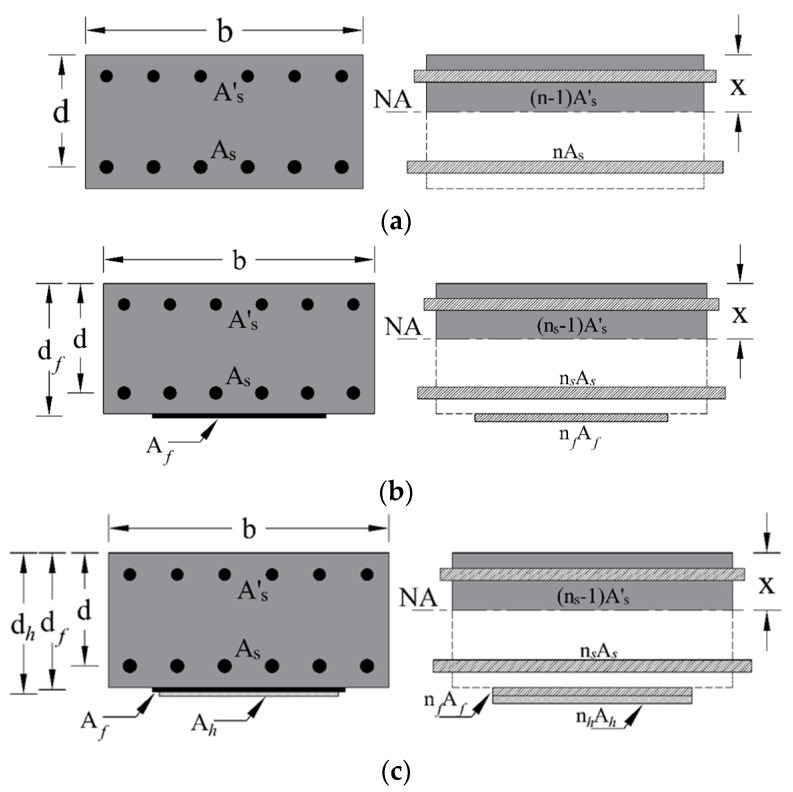
Gross and transformed sections for (**a**) control specimens; (**b**) WB-CON-S1; (**c**) WB-CON-S2.

For prismatic RC beams without cracking, the gross moment of inertia (MI), $I_{g}$, is deemed suitable for representing bending stiffness and estimating immediate deflections. In contrast, for cracked sections, the effective MI ($I_{e}$) must be utilized to achieve reliable and accurate deflection estimates.

(7) $$I_{e}={\left(\frac{M_{cr}}{M_{a}}\right)}^{3}I_{g}+\left[1-{\left(\frac{M_{cr}}{M_{a}}\right)}^{3}\right]I_{cr}\leq I_{g}$$

where $I_{cr}$ represents the MI of the cracked transformed section (refer to Figure 20), while $I_{g}$ denotes the MI of the gross cross-section about the centroidal axis. $M_{cr}$ is the cracking moment, and $M_{a}$ is the moment in the beam when deflection is evaluated. According to ACI 318 [45], the cracking moment *Mcr* is estimated from

(8) $$M_{cr}=\frac{f_{r}I_{g}}{y_{t}}$$

In this expression, $f_{r}$ represents concrete’s modulus of rupture. The term $y_{t}$ denotes the distance between the gross section’s centroidal axis and the extreme fiber tension. The factor λ is a modification coefficient that considers the reduced strength of lightweight concrete; ${f^{\prime }}_{c}$ refers to the concrete’s specified compression strength.

The elastic modulus of concrete $E_{c}$ was estimated using Equation (9) as per the ACI 318 provisions. The elastic modulus for steel $E_{s}$ was assumed to be 200,000 MPa. The MI of the cracked section $I_{cr}$ was calculated by transforming the steel reinforcement, following the procedures outlined in the ACI [52].

(9) $$E_{c}=4700\sqrt{{f^{\prime }}_{c}}$$

To analytically construct the load–deflection variation, the applied load was incrementally increased starting from zero in steps of 0.7 kN. Each load step was represented in terms of the corresponding moment Ma, allowing for the estimation of the effective MI, $I_{e}$, at that stage. If the computed $I_{e}$ exceeded the gross MI $I_{g}$, the latter was adopted for deflection estimation. The mid-span deflection δ of the simply supported beam under a central concentrated load was then calculated using the well-established Equation (10). In this equation, L represents the span length of the beam, P is applied load corresponding to the deflection being evaluated, and $I_{e}$ denotes the effective MI of the RC wide beam, respectively, as defined by Equations (9) and (7).

(10) $$\delta =\frac{PL^{3}}{48E_{c}I_{e}}$$

**Figure 21:**
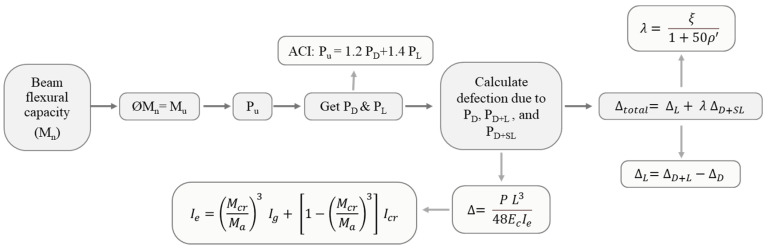
Total long-term deflection calculation.

Based on the ACI [52], the total long-term deflection was calculated from

(11) $$\delta_{total}=\delta_{L}+\lambda \;\delta_{D+SL}$$

where $\delta_{L}$ is the deflection corresponding to live load; λ is a long-term multiplier as per the ACI 318–19 code; $\delta_{D\;+\;SL}$ is the deflection corresponding to the dead and sustained live load. The sustained live load was taken as 25% of the total live load as used in the design of residential buildings. The analytical total long-term deflection is calculated as follows (see Figure 21).

Following the determination of the beam’s flexural capacity Mu, the corresponding ultimate load was computed. Subsequently, the dead and live loads were estimated by assuming the live load as a percentage of the dead load. Deflections were then calculated under dead load $\delta_{D}$, dead and live loads $\delta_{D\;+\;L}$, and dead and sustained live loads $\delta_{D\;+\;SL}$ using Equation (10). The long-term deflection multiplier λ was evaluated based on Equation (12).

(12) $$\lambda =\frac{\xi }{1+50\rho^{\prime }}$$

where ξ is the time reliant factor for sustained loads; $\rho^{\prime }$ is the rebar ratio for compression steel reinforcement.

The experimental total long-term displacement is calculated with Equation (11) as well. However, the values of the deflections due to dead load $\delta_{D}$, dead and live loads $\delta_{D\;+\;L}$, and dead and sustained live loads $\delta_{D\;+\;SL}$ are taken from the experimental load–deflection curve.

### Discussion of Analytical Results

**Figure 22:**
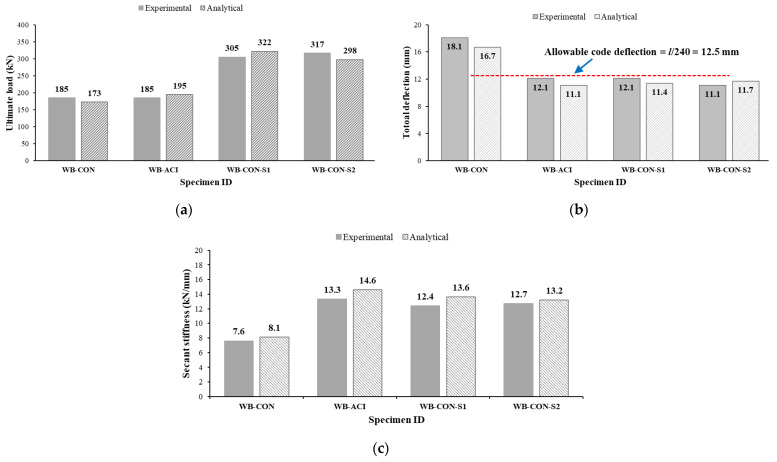
Comparison of experimental and analytical results of test specimens with regard to the following: (**a**) Peak load. (**b**) Total deflection. (**c**) Secant stiffness.

**Figure 23:**
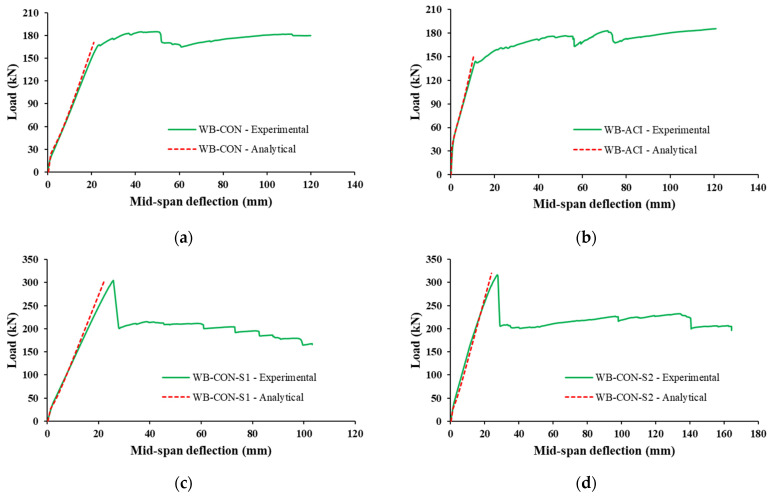
Analytical vs. experimental load–deflection curves for (**a**) WB-CON; (**b**) WB-ACI; (**c**) WB-CON-S1; (**d**) WB-CON-S2.

The aforementioned procedure was utilized to analytically compute the peak load and mid-span displacement values for both the control and retrofitted specimens investigated in this study. Table 7 and Figure 22 provide an exhaustive comparison of the experimental results with analytical results for total peak load, total deflection, and secant stiffness. Additionally, Figure 23 presents a comparison of the analytically derived load–deflection plots with the corresponding experimental curves, representing the immediate deflection behavior of the tested specimens. Table 7 reports total deflection as the sum of instantaneous live-load and long-term (dead and sustained live) deflections, accounting for time-dependent effects, as per the referenced codes [45,46]. Table 7 confirms the precision of analytical models in assessing the ultimate load capacity for all specimens, with deviations ranging from 0% to 7% for peak load. The calculated deflection values for both strengthened and unstrengthened beams closely matched their experimental counterparts, as evidenced by the results in Figure 22 and Figure 23 and Table 7. Moreover, the analytical models demonstrated reliable predictions of secant stiffness, with an error margin not exceeding 10%.

## 5. Conclusions

The test results of concrete wide beams supporting planted columns strengthened in flexure for deflection control with external CFRP sheets alone versus a hybrid system comprised of CFRP sheets along with CFRP/honeycomb plates were presented. The experimental findings were analyzed with respect to failure modes and the behavior noted in the load–deflection responses. Furthermore, the test outcomes were critically compared with predictions of the analytical models developed for estimating the flexural capacity and deflection performance of the specimens. The key outcomes of this investigation are summarized below:

- The control beams exhibited the typical flexure failure characteristic of tension-controlled reinforced concrete elements. This included the growth of major flexure cracks near the mid-span, yielding of tension reinforcement, and subsequent concrete crushing at the top (compression zone) surrounding the planted column. In contrast, the failure sequence of the retrofitted beams initiated with the tension rebars’ yielding, then debonding of the CFRP reinforcement, ultimately culminating in the crushing of concrete within the compression region.
- Both strengthening techniques employed in this study proved to be beneficial in upgrading the flexure performance of concrete wide beams, particularly causing enhancement in strength as well as stiffness. Both upgrading schemes significantly reduced the total deflection of beams to a level that satisfied the serviceability requirements of the current codes.
- The inclusion of CFRP reinforcement alone in the specimen WB-CON-S1 resulted in a 65% enhancement in peak load and a 63% enhancement in stiffness in comparison with the control beam WB-CON. The stiffness of WB-CON-S1 was also comparable to that of the ideal control specimen WB-ACI, with only a 6% difference. Additionally, the specimen exhibited a 3% increase in dissipated energy relative to WB-CON; however, this value was 1% lower than that of WB-ACI.
- The adoption of a hybrid strengthening system comprising CFRP sheets and CFRP/honeycomb plates in the specimen WB-CON-S2 led to increases of 71% and 67% in the peak load and stiffness, respectively, relative to the control beam WB-CON. The stiffness of WB-CON-S2 was also closely aligned with that of the ideal control specimen WB-ACI, with only a 4% difference. In addition, the hybrid system significantly improved energy dissipation, showing increases of 80% and 74% in comparison with WB-CON and WB-ACI, respectively.
- Analytical models were presented in this study to estimate the flexural capacity and deflection of both control and strengthened reinforced concrete wide beams. These models demonstrated strong predictive capability, with maximum errors of 7% for peak load and 8% for deflection when compared to the test results.
- A key limitation of the study is the testing of only one specimen per configuration, preventing statistical evaluation of variability. While the responses align with established trends, the restricted sample size limits generality; future studies with multiple replicates and parametric variations are recommended to improve statistical reliability. Additionally, future studies could incorporate full-scale tests or finite element modeling to complement the experiments and enable parametric exploration of RC wide beams upgraded in flexure using FRP and/or honeycomb composites.

## Figures and Tables

**Table 1:** Test matrix.

Specimen ID	Description	No. of Specimens
WB-CON	Control specimen Meets code flexural requirements Meets code shear requirements Does not meet code deflection requirements Total deflection (considering long-term effect) = 16.7 mm > l/240 (= 12.5 mm)	1
WB-ACI	Control specimen Meets code flexural requirements Meets code shear requirements Meets code deflection requirements Total deflection (considering long-term effect) = 11.1 mm < l/240 (= 12.5 mm)	1
WB-CON-S1	Strengthened specimen Has the same design as the control beam (WB-CON) but strengthened on the tension side using 4.5 layers of CFRP sheets Total deflection (considering long-term effect) = 11.4 mm < l/240 (= 12.5 mm)	1
WB-CON-S2	Strengthened specimen Has the same design as the control beam (WB-CON) but strengthened on the tension side using one layer of CFRP sheet combined with CFRP/honeycomb plate Total deflection (considering long-term effect) = 11.7 mm < l/240 (= 12.5 mm)	1
Total No. of Specimens		4

**Table 2:** Deflection calculation for test specimens *.

Specimen	Mid-Span Deflection Due to				Total Deflection (δ_total_ = δ_L_ + λδ_D+SL_) (mm)	Allowable Deflection (mm)	Deflection Check
	Dead Load (δ_D_) (mm)	Dead and Live Loads (δ_D+L_) (mm)	Live Load (δ_L_) (mm)	Dead and Sustained Live Loads (δ_D+SL_) (mm)			
WB-CON	9.3	13.4	4.1	10.4	16.7	12.5	Not good
WB-ACI	5.9	8.5	2.6	6.6	11.1	12.5	OK
WB-CON-S1	6.5	9.2	2.7	7.2	11.4	12.5	OK
WB-CON-S2	6.7	9.4	2.7	7.3	11.7	12.5	OK

* λ = long-term multiplier as per ACI 318–19 code.

**Table 3:** Tensile properties of steel bars.

Bar Diameter (mm)	Tensile Strength (MPa)	
	Yield	Ultimate
8	525	537
10	547	572
12	550	657
18	528	658

**Table 4:** Mechanical properties of the used strengthening materials.

Material	CFRP Sheet *	CFRP Honeycomb Plate *	Epoxy Adhesive Mortar (SIKA-31/41) **	HM-180C3P Carbon Fiber Impregnated Adhesive **
Elastic modulus (GPa)	71.46	90	2.6	3.1
Tensile strength (MPa)	710	1100	13	60
Rupture strain	0.01	0.012	-	-
Thickness per CFRP layer (mm)	1.0	0.65	-	-
Thickness of honeycomb core (mm)	-	11.7	-	-
Compressive strength (MPa)	-	-	52	95
Bond strength to concrete (MPa)	-	-	>4	>2.5

* Based on testing of standard coupons. ** Based on manufacturer’s datasheet.

**Table 5:** Experimental results of test specimens.

Specimen	Cracking Load (kN)	Yield Load (kN)	Peak Load (kN)	Mid-Span Deflection (mm)				Mode of Failure *
				At Cracking Load	At Yield Load	At Peak Load	At Ultimate State	
WB-CON	21.4	140.7	185.4	1.6	18.5	49.6	119.8	Y-C
WB-ACI	54.9	137.7	185.3	2.0	10.3	71.4	120.7	Y-C
WB-CON-S1	31.9	254.7	305.1	1.7	20.6	25.7	26.9	Y-IC
WB-CON-S2	35.2	269.8	316.6	1.7	21.2	27.3	24.2	Y-IC

* Y-C: Yielding of main tension steel bars followed by concrete crushing. Y-IC: Yielding of main tension steel bars followed by IC debonding of CFRP sheet/honeycomb.

**Table 6:** Evaluated parameters for test specimens.

Specimen	Pre-Cracking Stiffness (kN/mm)	Secant Stiffness (kN/mm)	Post-Cracking Stiffness (kN/mm)	Dissipated Energy (kN·m)	Deflection Ductility
WB-CON	13.4	7.6	7.1	19.2	6.4
WB-ACI	27.3	13.3	10.0	19.9	11.7
WB-CON-S1	18.8	12.4	11.8	19.7	5.0
WB-CON-S2	20.5	12.7	12.1	34.6	7.8

**Table 7:** Comparison of analytical with experimental results of tested specimens *.

Specimen	Experimental			Analytical			Difference (%)		
	Peak Load (kN)	Total Deflection (mm)	Secant Stiffness (kN/mm)	Peak Load (kN)	Total Deflection (mm)	Secant Stiffness (kN/mm)	Peak Load	Total Deflection	Secant Stiffness
WB-CON	185.4	18.1	7.6	173.3	16.7	8.1	6.5	7.6	6.6
WB-ACI	185.3	12.1	13.3	194.5	11.1	14.6	5.0	7.6	9.7
WB-CON-S1	305.1	12.1	12.4	322.2	11.4	13.6	5.6	5.8	9.6
WB-CON-S2	316.6	11.1	12.7	297.8	11.7	13.2	5.9	5.4	3.9

* δ_total_ = δ_L_ + λδ_D+SL_; δ_total_ = total deflection; δ_L_ = deflection due to live load; λ = long-term multiplier as per ACI code; δ_D+SL_ = deflection due to dead and sustained live loads.

## Data Availability

The data presented in this study are available on request from the corresponding author.
